# How the *JCI’s* most-cited paper sparked the field of lipoprotein research

**DOI:** 10.1172/JCI177475

**Published:** 2024-02-15

**Authors:** Michael S. Brown, Joseph L. Goldstein

**Affiliations:** Department of Molecular Genetics, University of Texas Southwestern Medical Center, Dallas, Texas, USA.

A profound medical triumph of the last half of the 20th century was the elucidation of the transport system mediated by plasma lipoproteins. Scientists identified the proteins that carry water-insoluble lipids in the plasma, the enzymes and receptors that remove these lipids, and the separate roles that each lipoprotein plays in physiology and disease. Genetic and environmental abnormalities of lipoprotein metabolism were defined, and powerful treatments were developed and instituted.

The *JCI* played a major role in the lipoprotein transport story. The *JCI*’s publication of a seminal 1955 paper that outlined a method by which lipoproteins could be separated from other plasma proteins in quantities that permit structural and functional characterization was crucial (1). The paper was entitled, “The distribution and chemical composition of ultracentrifugally separated lipoproteins in human serum” (Figure 1A). Its three young authors were Richard Havel, Howard Eder, and Joseph Bragdon, all of whom worked at the National Heart Institute at the NIH in Bethesda, Maryland. The impact of their paper is reflected in the number of times it has been cited — no fewer than 8,913 times. Figure 1B shows the annual citations, which peaked when lipoprotein research was most intense in the late 1990s and declined thereafter when the major questions had been answered. The 8,913 citations are the most for any of the 31,282 papers published in the 100-year history of the *JCI*. The citations are testimony to the universal and enduring use of the Havel-Eder-Bragdon method in nearly all papers devoted to lipoprotein structure and metabolism. Figure 1C shows a photograph of the late Richard J. Havel, the paper’s lead author.

Lipoproteins are spherical particles with a core of nonpolar lipids, primarily triglycerides and cholesterol esters. The core is surrounded by a phospholipid coat that acts as a detergent to solubilize the particle. Each class of lipoproteins has a distinct complement of proteins that surround the lipid core. Inasmuch as lipids are less dense than water, lipoprotein particles float when subjected to ultracentrifugation. Each class of lipoproteins floats at a different density depending on the ratio of core lipids to proteins.

## The dawn of lipoprotein research

The modern era of lipoprotein research began in 1949 when John Gofman and colleagues published a paper in which they used a new instrument called the analytical ultracentrifuge to classify serum (or plasma) lipoproteins according to their flotation properties (2). They were able to define triglyceride-rich lipoproteins because they were the lightest. They identified 2 classes of cholesterol-rich lipoproteins, one corresponding to the particles we now call low-density lipoprotein (LDL) and the other identified as high-density lipoprotein (HDL). In subsequent publications, Gofman and his associates demonstrated that cholesterol-fed rabbits and patients with heart attacks had elevations primarily in the LDL fraction (3, 4). Their studies were the first to identify LDL specifically, in contrast to total plasma cholesterol, as a marker for atherosclerosis.

Havel and his coworkers accepted Gofman’s results, but they realized that the analytical ultracentrifuge would not permit further studies of lipoproteins (5). The analytical ultracentrifuge measured the flotation rates of small amounts of lipoproteins depending on their density. The instruments were expensive, and they were not suitable for preparation of large amounts of lipoproteins that would be necessary for biochemical characterization. The field needed a new approach.

In their classic 1955 paper, Havel, Eder, and Bragdon devised a method to isolate large amounts of lipoproteins using a preparative ultracentrifuge (1). Aliquots of plasma were placed in the bottom of ultracentrifuge tubes, and the density of the plasma was adjusted by addition of a solution containing sodium chloride and concentrated potassium bromide. The plasma sample was overlaid with a solution of the same density. After ultracentrifugation for 20 hours, the particles that were lighter than the adjusted density floated to the top of the centrifuge tube, and there was a clear layer separating the lipoproteins from the infranatant plasma. The next step was to take the infranatant plasma and raise the density further by addition of a larger amount of the sodium chloride/potassium bromide solution. Again, the tube was subject to ultracentrifugation, and the lipoproteins that floated at that density were obtained.

Through experimentation at different densities, the Havel group was able to isolate lipoprotein fractions that we now know as LDL and HDL. They used their method to measure the amount of cholesterol contained in each one of these fractions in 43 healthy young men and women, thereby defining the normal range. They showed that men tended to have higher LDL levels, and women had higher HDL. They also studied patients with atherosclerosis or diseases that predispose to it. They showed that the LDL fraction was elevated selectively in these individuals.

The Havel method was described in precise detail in their paper (1, 6). The method was easy to follow for anyone with a preparative ultracentrifuge, which was becoming a standard laboratory instrument in the 1950s. For the first time, scientists were able to isolate amounts of lipoproteins sufficient to permit identification and classification of the protein components. New techniques of protein chemistry were being developed at this time, and they allowed the protein components to be isolated and classified based on their NH_2_-terminal amino acids.

## Lipoprotein research through the decades

When the two of us began our work on LDL in 1973, we used the Havel method to isolate the LDL that we added to the culture medium of human fibroblasts (7, 8). We attached a radioactive ^125-^I tag to the LDL and demonstrated that cells have high-affinity cell surface LDL receptors that use LDL as a source of cholesterol. We discovered that patients with homozygous familial hypercholesterolemia (FH) lack LDL receptors (7–9). The Havel method was indispensable for this work.

In the 1990s, the *JCI* published other highly cited papers dealing with the role of lipoproteins in the atherosclerosis process itself. One of these papers (1,279 citations; Web of Science) described the first mouse model of homozygous FH. By 1993, our laboratory had isolated the gene for the LDL receptor, and we used the methods described by others to inactivate the LDL receptor gene in the mouse (10). Destruction of the LDL receptor gene (*Ldlr^–/–^*) led to massive hypercholesterolemia, owing to a reduced rate of removal of the lipoprotein from plasma. The hypercholesterolemia in the *Ldlr^–/–^* mice could be reversed by delivering an adenovirus encoding the LDL receptor gene to the liver.

**Figure 1:**
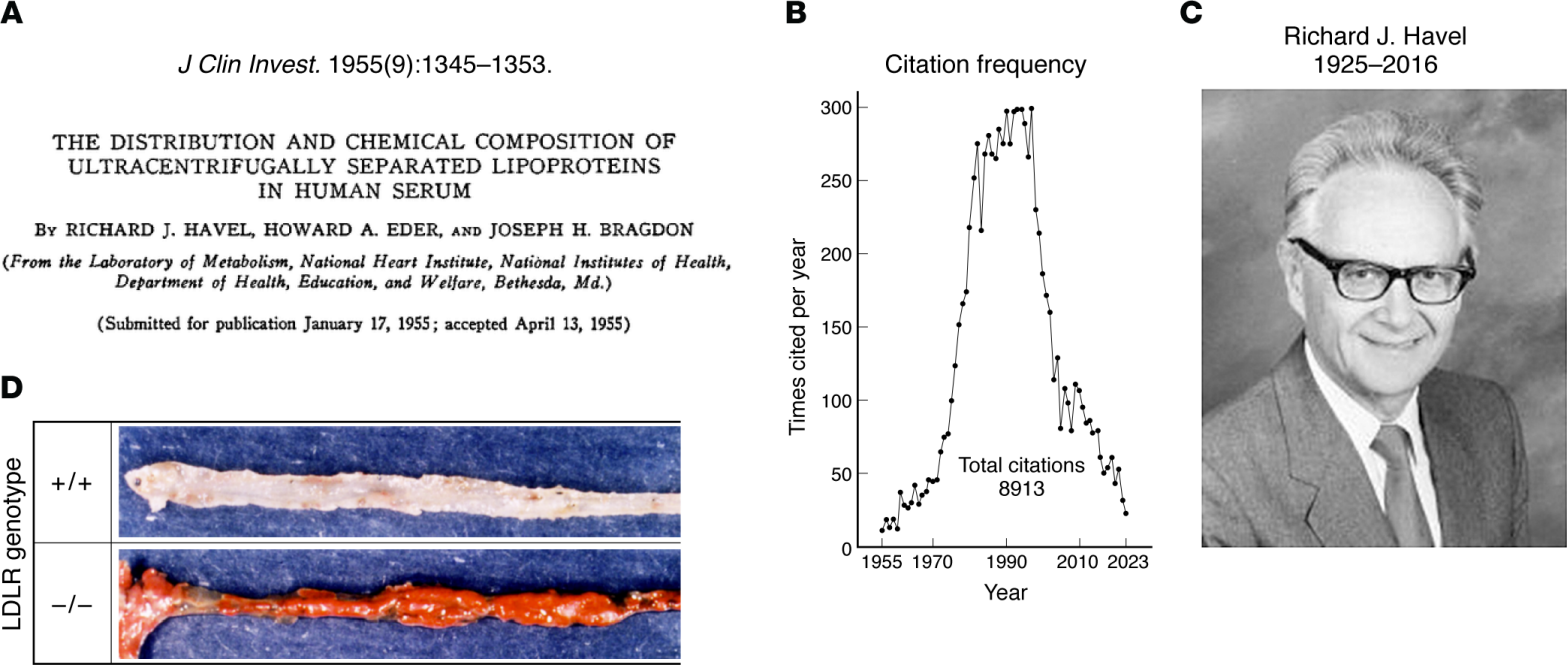
The most-cited paper in the 100-year history of the *JCI*. (**A**) A copy of the title page of the 1955 paper by Havel, Eder, and Bragdon, who described a method that used the ultracentrifuge to isolate plasma lipoproteins. (**B**) Annual citations from 1955 to 2023. The total number of citations is 8,913, according to data from the Web of Science. Google Scholar, whose citation counts are based on a larger set of publications, including non-peer-reviewed articles and book chapters, lists the total number of citations at 10,012). (**C**) The late Richard J. Havel, the lead author of the paper. The photograph is reproduced from Kane and Malloy (12). (**D**) Photographs of the luminal surface of aortas from a cholesterol-fed, wild-type mouse (top) and an *Ldlr^–/–^* mouse (bottom). The aortas were stained for lipid with Sudan IV. Mice from both genotypes were fed a diet containing 1.25% cholesterol, 7.5% cocoa butter, 7.5% casein, and 0.5% sodium cholate for 8 months and were sacrificed at 13 months. Photographs are reproduced from Ishibashi et al. (11).

Another highly cited *JCI* paper (571 citations) described the massive xanthomatosis and atherosclerosis that occurred when the *Ldlr^–/–^* mice were fed a high-cholesterol diet (11). Wild-type mice showed a minimal elevation in LDL when fed this same diet (total cholesterol of 130–150 mg/dL). As a result, wild-mice mice were not useful for studies of the atherosclerotic process. This *JCI* paper showed that the LDL receptor is necessary for this dietary resistance. When the *Ldlr^–/–^* mice were fed a high-cholesterol diet, they developed massive hypercholesterolemia, with cholesterol levels of 1,600–2,000 mg/dL. After 8 months on this diet, the animals showed all of the pathologic features of homozygous FH. They exhibited visible cholesterol deposits in the tendons and around the eyes, mimicking tendon xanthomas and xanthelasmas. Most important, their major blood vessels showed diffuse atherosclerosis. Figure 1D shows the aorta of an *Ldlr^–/–^* mouse that was opened and treated with a stain that colors lipid deposits red. Histologically, these deposits showed all of the features seen in human atherosclerotic plaques.

The *Ldlr^–/–^* mice have proven to be a useful model for studies of the pathogenesis of atherosclerotic plaques. The mutant mice were deposited at The Jackson Laboratory 30 years ago, where they have been one of the most highly demanded strains of genetically engineered mice. An average of approximately 8,000 *Ldlr^–/–^* mice are sent each year to around 90 institutions throughout the world.

## Concluding remarks

As it celebrates its 100th anniversary, we salute the *JCI* and hundreds of editors who took time from their busy academic careers in order to select the strongest papers for publication. The *JCI* has played a central role at the interface between basic biomedical science and the clinical problems that benefit from it. We look forward to the next 100 years.

## Version 1 02/15/2024

Electronic publication
